# *FOXC2* Disease Mutations Identified in Lymphedema Distichiasis Patients Impair Transcriptional Activity and Cell Proliferation

**DOI:** 10.3390/ijms21145112

**Published:** 2020-07-20

**Authors:** Daniela Tavian, Sara Missaglia, Sandro Michelini, Paolo Enrico Maltese, Elena Manara, Alvaro Mordente, Matteo Bertelli

**Affiliations:** 1 Laboratory of Cellular Biochemistry and Molecular Biology, CRIBENS, Università Cattolica del Sacro Cuore, 20145 Milan, Italy; sara.missaglia@unicatt.it; 2 Psychology Department, Università Cattolica del Sacro Cuore, 20123 Milan, Italy; 3 Department of Vascular Rehabilitation, San Giovanni Battista Hospital, 00148 Rome, Italy; s.michelini@acismom.it; 4 Laboratory of Molecular Genetics, International Association of Medical Genetics, MAGI’s Lab s.r.l., 38068 Rovereto, Italy; paolo.maltese@assomagi.org (P.E.M.); matteo.bertelli@assomagi.org (M.B.); 5 MAGI EUREGIO, 39100 Bolzano, Italy; elena.manara@assomagi.org; 6 Dipartimento di Scienze di Laboratorio ed Infettivologiche, Fondazione Policlinico Universitario A. Gemelli, IRCCS, 00168 Rome, Italy; Alvaro.Mordente@unicatt.it; 7 Facoltà di Scienze della Formazione, Università Cattolica del Sacro Cuore, 20123 Milan, Italy

**Keywords:** lymphedema distichiasis, FOXC2, nuclear transcription factor, functional analysis, transactivation activity, cell death, nonsense-mediated decay

## Abstract

*FOXC2* is a member of the human forkhead-box gene family and encodes a regulatory transcription factor. Mutations in *FOXC2* have been associated with lymphedema distichiasis (LD), an autosomal dominant disorder that primarily affects the limbs. Most patients also show extra eyelashes, a condition known as distichiasis. We previously reported genetic and clinical findings in six unrelated families with LD. Half the patients showed missense mutations, two carried frameshift mutations and a stop mutation was identified in a last patient. Here we analyzed the subcellular localization and transactivation activity of the mutant proteins, showing that all but one (p.Y109*) localized to the nucleus. A significant reduction of transactivation activity was observed in four mutants (p.L80F, p.H199Pfs*264, p.I213Tfs*18, p.Y109*) compared with wild type FOXC2 protein, while only a partial loss of function was associated with p.V228M. The mutant p.I213V showed a very slight increase of transactivation activity. Finally, immunofluorescence analysis revealed that some mutants were sequestered into nuclear aggregates and caused a reduction of cell viability. This study offers new insights into the effect of *FOXC2* mutations on protein function and shows the involvement of aberrant aggregation of FOXC2 proteins in cell death.

## 1. Introduction

The forkhead transcription factor C2 (FOXC2) plays a key role in regulation of lymphatic endothelial cell differentiation, formation of smooth muscle cell layers and morphogenesis of lymphatic valves during embryogenesis. Dominant mutations in the *FOXC2* gene (MIM *602402) are linked to lymphedema-distichiasis (LD) syndrome (MIM #153400) and cause pleiotropic effects in different types of tissue [1,2,3]. Indeed, the syndrome is characterized by variable age of onset and clinical findings, including extradural cysts, heart defects, cleft palate, fetal cystic hygroma and hydrops [4,5,6]. Recent studies have demonstrated that FOXC2 is also a crucial regulator of several hallmarks of cancer progression [7,8,9,10]. In tumor cells, FOXC2 overexpression has been shown to promote proliferation, epithelial-mesenchymal transition (EMT), metastases and drug resistance [11,12,13,14]. In vascular endothelial cells, FOXC2 promotes expression of multiple genes that enhance angiogenesis [15,16]. Despite the significant involvement of FOXC2 in many oncogenic functions, much remains to be clarified regarding the molecular mechanisms of their induction.

**Figure 1:**
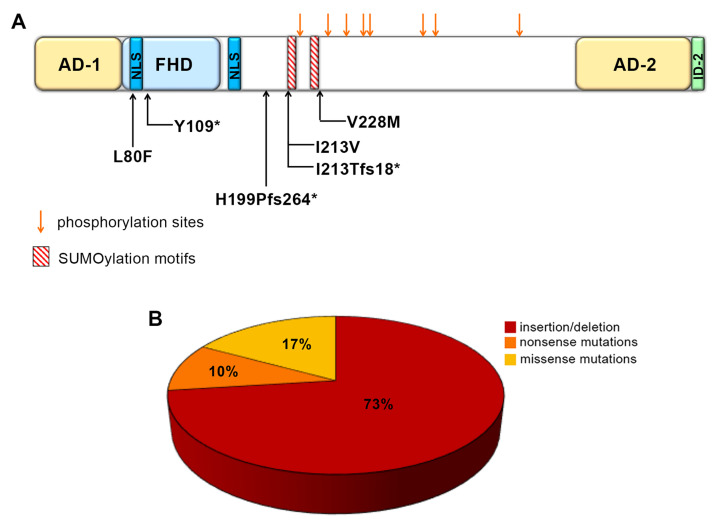
Structural domains of FOXC2 protein and FOXC2 gene mutations. (**A**) In the schematic representation of FOXC2 (amino acids 1–501), activation domain 1 (AD-1) is between amino acids 1 and 70. The forkhead domain (FHD, amino acids 71–162) is the DNA-binding region and also contains nuclear signal 1 (NLS1, amino acids 78–93). The central region (amino acids 163–394) includes NLS2, located between amino acids 168 and 176, two SUMOylation motifs and eight phosphorylation sites. The C-terminal region includes activation domain 2 (AD-2, amino acids 395–494) and inhibitory domain 2 (ID-2, amino acids 494–501). The sites of the mutations analyzed in this study are indicated in the scheme. (**B**) Percentages of different FOXC2 mutations identified in lymphedema-distichiasis patients.

The FOXC2 gene at 16q24.3 produces a transcript from a 1.5-kb single exon coding region. The 501 amino acid FOXC2 protein contains: (i) a transactivation domain 1 (AD-1) extending from the first amino acid to the forkhead DNA binding domain (FHD, amino acid 71); (ii) the FHD (amino acids 71 to 162) with nuclear localization signal 1 (NLS1, amino acids 78-93) [17]; (iii) a central region, where the NLS2 sequence (amino acids 168-176) and some conserved phosphorylation and SUMOylation sites were recently identified, conferring a negative regulative role to this sequence [18,19]; (iv) a C-terminal sequence, consisting of a second transactivation domain (AD-2, amino acids 395-494) and an inhibitory region (ID2, amino acids 495-501) (Figure 1A) [18,20].

The majority of FOXC2 mutations linked to lymphedema-distichiasis syndrome are insertion/deletion (73%) and nonsense mutations (10%) (Figure 1B) [2,21,22,23]. In most cases, they are presumed to cause loss of the gene product because their mRNAs contain a premature termination codon (PTC) and are thus eliminated through the nonsense-mediated decay (NMD) pathway [24]. However, in the specific case of FOXC2, NMD should only be considered a theoretical hypothesis, because no data is available on the lack of FOXC2 mutated mRNAs or proteins in different biological tissues of affected subjects. In a small percentage of patients (1–2%), complex rearrangements have also been found, leading to complete loss of protein expression. Finally, almost 15–17% of LD patients carry FOXC2 missense mutations scattered along the coding sequence (www.hgmd.cf.ac.uk) [23,25]. A few have been investigated functionally by luciferase assay [26,27]. Those located in the forkhead domain impair DNA-binding and transcriptional activation [17,26], while those located outside the forkhead domain can cause loss or gain of function [27].

It may be supposed that the pathogenesis of LD is mostly associated with FOXC2 haploinsufficiency. However in a restricted number of patients, a gain in FOXC2 function has been observed and demonstrated to be equally harmful [26,27]. The molecular mechanisms underlying lymphatic system damage have yet to be explained, and the functional role of most of the mutations identified in LD patients remains largely unexplored.

In this study, we performed the functional characterization of different types of mutations identified in six unrelated families with LD to obtain insights into structure-function relationships of FOXC2 transcription factor. We also explored the involvement of some FOXC2 mutant proteins in cell death.

## 2. Results

### 2.1. Mutation Analysis 

The clinical features of the patients included in our study have already been reported [23]. The main molecular and clinical findings are summarized in Table 1. Briefly, all patients showed lymphedema of the lower limbs, varying widely in degree. Half of them also had distichiasis. No evidence of heart defects, cleft palate, extradural cysts or other distinctive features were reported, except for varicose veins, cellulitis and spinal extradural cysts in Patient 2, and varicose veins, cardiac arrhythmia and upslanting toenails in Patient 3. No superficial or deep venous insufficiency or recurrent erysipelas was observed.

### 2.2. In Vitro Characterization of FOXC2 Mutations 

To determine whether the different mutations affected FOXC2 function, altering nuclear localization or impairing transactivation activity, we subcloned wild type FOXC2 cDNA into a pcDNA3.1/NT-GFP-TOPO expression vector and performed specific site-directed mutagenesis. Then we transfected HeLa cells with FOXC2-GFP mutant plasmids and performed Western blot, immunofluorescence and transactivation analysis. 

**Figure 2:**
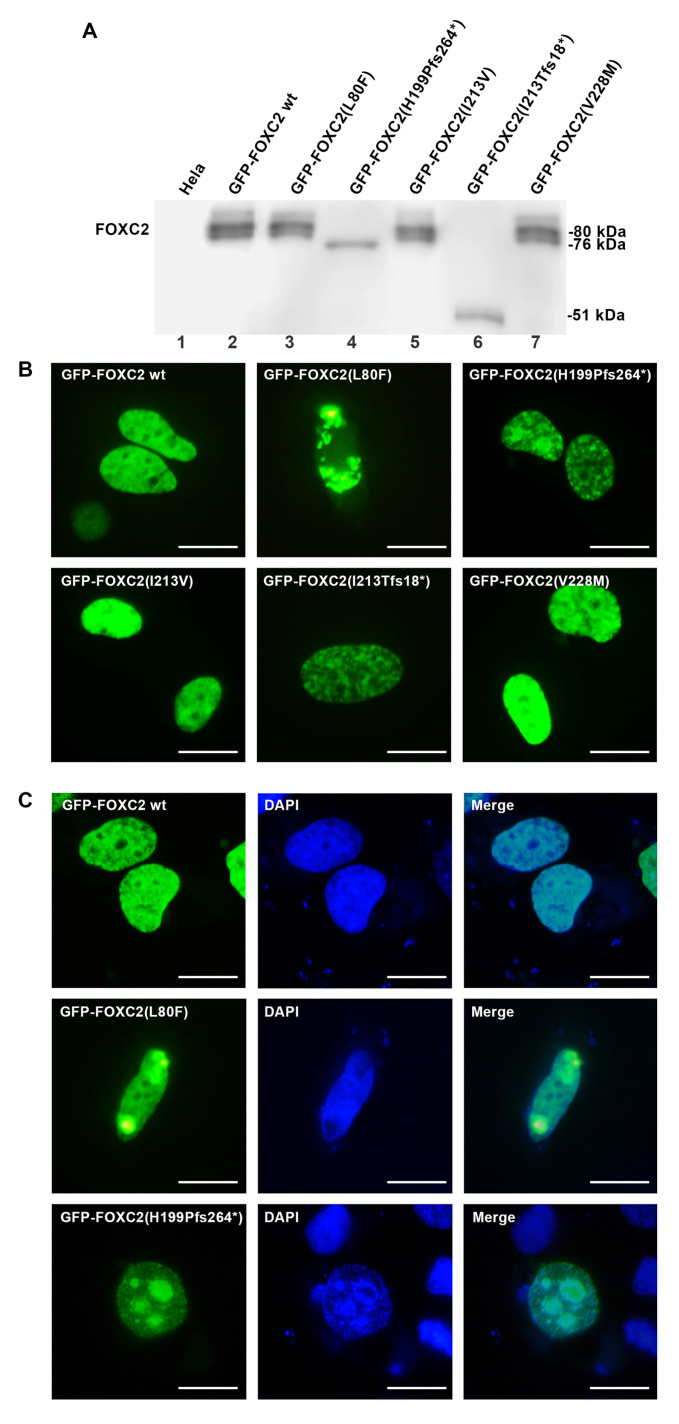
FOXC2 protein expression of mutant recombinant plasmids. (**A**) Western blot analysis of exogenous FOXC2 protein expression in HeLa cells transfected with wild type and mutant plasmids (pFOXC2-GFP; *pFOXC2*(L80F)-GFP; pFOXC2(H199Pfs*264)-GFP; pFOXC*2*(I213V)-GFP; pFOXC2(I213Tfs*18)-GFP; pFOXC2(V228M)-GFP); lane 1 referred to nontransfected HeLa cells; (**B**) Immunofluorescence detection of FOXC2 mutant proteins in HeLa cells after transient transfection. HeLa cells were transfected with one of the following plasmids: pFOXC*2*-GFP, pFOXC2(L80F)-GFP; pFOXC2(H199Pfs*264)-GFP; pFOXC2(I213V)-GFP; pFOXC2(I213Tfs*18)-GFP; pFOXC2(V228M)-GFP) and fixed with 3% paraformaldehyde 24 h later. The nuclear localization of all FOXC2 mutant proteins was detected by direct immunofluorescence analysis of plasmids tagged with GFP (in green); 40× magnification. Scale bar: 10 μm. The frameshift and pL80F mutations gave rise to nuclear FOXC2 protein aggregates; (**C**) Immunofluorescence evaluation of HeLa cells transiently transfected with *pFOXC2*-GFP, pFOXC2(L80F)-GFP; pFOXC2(H199Pfs*264)-GFP. Mutations p.L80F and p.H199Pfs*264 determined abnormal protein assembly. In green, FOXC2 proteins tagged with GFP and in blue cell nuclei stained with DAPI; 40× magnification. Scale bar: 10 μm.

**Figure 3:**
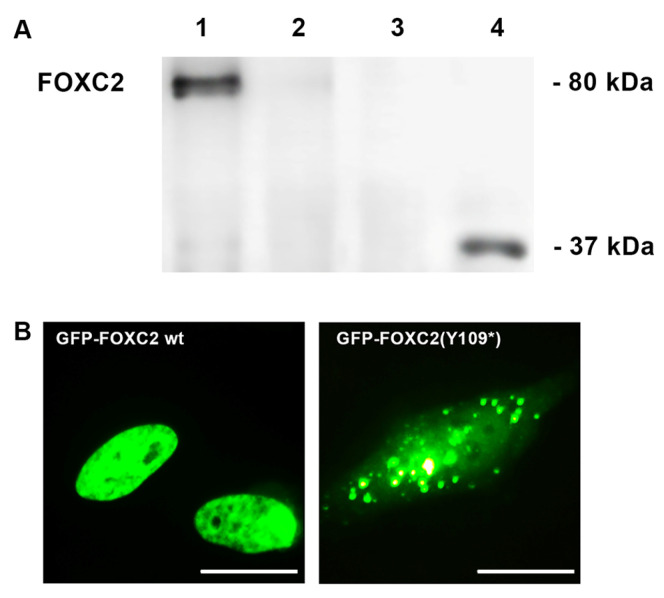
Exogenous expression of p(Y109*) FOXC2 mutant protein. (**A**) Western blot analysis of exogenous FOXC2 protein expression from nuclear (lanes 1 and 2) and cytoplasmic (lanes 3 and 4) protein extracts of HeLa cells transfected with control and pFOXC2(Y109*)-GFP mutant plasmid; (**B**) immunofluorescence detection of control (nuclear) and p.Y109* (cytoplasmic) FOXC2 protein in HeLa cells 24 h after transfection. FOXC2 proteins were localized by direct immunofluorescence analysis of plasmids tagged with GFP (in green); 40× magnification. Scale bar: 10 μm.

Immunoblot detection of nuclear extracts allowed identification of a FOXC2 protein of almost 80 kDa from transfection of wild type (*pFOXC2-GFP*) and missense mutant plasmids (pFOXC2(L80F)-GFP; pFOXC2(I213V)-GFP; pFOXC2(V228M)-GFP) (Figure 2A, lanes 3, 5 and 7). Instead, two truncated proteins of 76 and 51 kDa were detected after pFOXC2(H199Pfs*264)-GFP and pFOXC2(I213Tfs*18)-GFP transfection, respectively (Figure 2A, lanes 4 and 6). Western blot analysis showed similar stable expression of all FOXC2 nuclear recombinant proteins (Figure 2A). Unlike other mutations, p.Y109* was only detected in cytoplasmic extracts of transfected cells as a truncated protein of 37 kDa (Figure 3A, lane 4). The missense and the wild type FOXC2 proteins showed a typical phosphorylation pattern with multiple immunoreactive bands. On the contrary, the p.H199Pfs*264 and p.Y109* mutations gave rise to single bands, having lost the phosphorylation sites in the central region of FOXC2 protein (Figure 2A, lanes 4; Figure 3A lane 4). The I213Tfs*18 mutation also lacked the canonical phosphorylation sites of FOXC2 protein. However it contained some new sites in the amino acid sequence due to frameshift (Figure 2A, lane 6).

We then investigated whether the subcellular localization of the FOXC2 mutant proteins was affected. Immunofluorescence analysis revealed a homogeneous nuclear distribution of wild type FOXC2 protein (Figure 2B). None of the disease mutations identified in our patients affected nuclear localization except p.Y109* (Figure 3B). In agreement with the Western blot results (Figure 3A, lane 4), this mutant showed cytoplasmic staining and protein aggregates (Figure 3B). The p.H199Pfs*264 and p.I213Tfs*18 FOXC2 mutants caused production of nuclear aggregates (Figure 2C). In our system, the p.L80F FOXC2 mutant protein also induced intranuclear protein aggregation (Figure 2C). The last two mutant proteins, p.I213V and p.V228M, showed a homogeneous fluorescent signal like the wild type FOXC2 (Figure 2B). Expression vectors without TAG were used to transfect HeLa cells with wild type, frameshift and missense FOXC2 sequences; immunofluorescence analysis performed using FOXC2 antibody revealed comparable data to those obtained transfecting GFP-FOXC2 proteins (Supplementary Figure S1). 

**Figure 4:**
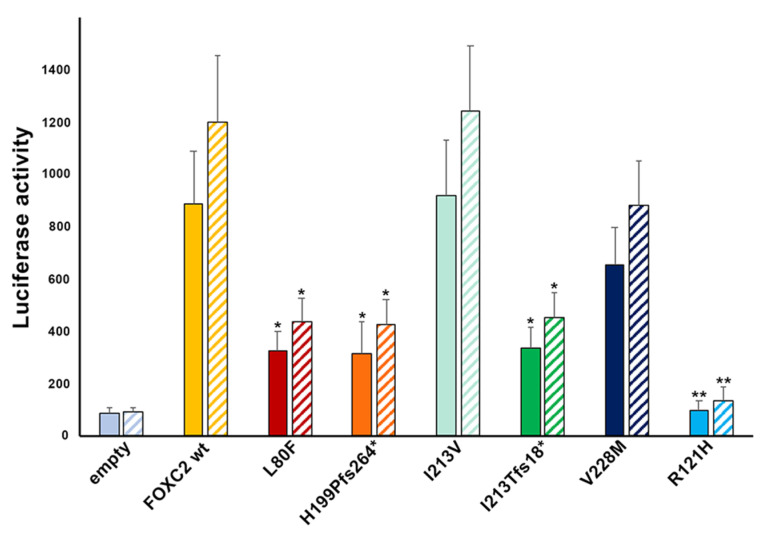
Transactivation activity of control and mutant FOXC2 proteins. Luciferase assays were used to measure protein transactivation. Transactivation activity of control and mutant FOXC2-GFP fusion proteins (squares with uniform colors) and mutant FOXC2 proteins without any tag (bars with diagonal stripes), compared with that of empty vector in HeLa cells, is indicated in fluorescence units (vertical axis). Thick bar: mean value, error bar: SD. Significant differences were detected by Student’s *t*-test. *p*-values <0.05 and <0.01 were considered significant and indicated * and **, respectively.

Finally, we investigated the ability of mutant proteins to activate a luciferase reporter vector. The p.R121H mutation was included as negative control in our experiment because it was previously tested in a luciferase assay and showed total loss of function [17,27]. Mutant proteins p.L80F, p.H199Pfs*264 and p.I213Tfs*18 activated the luciferase reporter vector by 30, 28 and 36%, respectively, compared to wild type FOXC2 protein (Figure 4; *p* = 0.04, *p* = 0.045, *p* = 0.047). These mutations impaired more than 50% of FOXC2 activity. The p.V228M mutation also decreased transcription activity, but only by 30% with respect to FOXC2 wild type (*p* = 0.11). Instead, the I213V mutation showed transcription activity slightly higher than that of the FOXC2 control protein (Figure 4; *p* = 0.9). The p.Y109* mutant protein was excluded from this set of experiments since it was previously demonstrated as unable to localize in the nucleus (Figure 3B). GFP-FOXC2 fusion proteins recapitulated transcriptional activity of FOXC2 mutant proteins without TAG.

### 2.3. Cell Death Induction of Mutant FOXC2 Proteins

**Figure 5:**
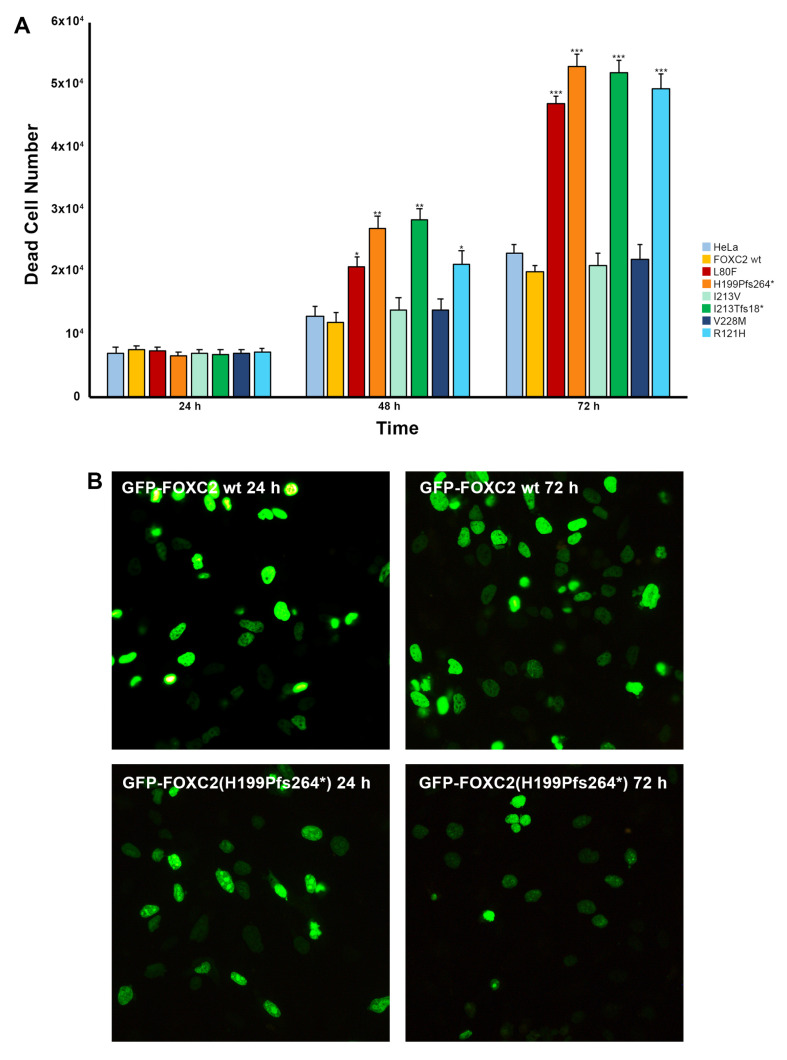
Viability analysis of HeLa cells transfected with native and mutant FOXC2-GFP constructs. (**A**) Statistical analysis of cell death in HeLa cells 24, 48 and 72 h after transient transfection with control and mutant FOXC2-GFP plasmids: average ± SD of at least three independent experiments. Statistical analysis was performed using the two-tailed Student’s *t*-test. * *p* < 0.05, ** *p* < 0.01, *** *p* = 0.000; (**B**) immunofluorescence analysis of pFOXC2-GFP and pFOXC*2*(H199Pfs*264)-GFP plasmids 24 and 72 h after transient transfection in HeLa cells. Nuclear localization of FOXC2 proteins was detected by direct immunofluorescence analysis of plasmids tagged with GFP (in green); 20× magnification.

Observation of abnormal nuclear aggregation of some FOXC2 mutant proteins led us to speculate that it could induce cell death. Therefore, wild type and FOXC2 mutant plasmids were used to perform HeLa transfection experiments and cell death was monitored by trypan blue staining at various times (24, 48 and 72 h) after transfection. As shown in Figure 5A, p.L80F, p.H199Pfs*264 and p.I213Tfs*18 FOXC2 proteins significantly induced cell death at 48 and 72 h (48 h: *p* = 0.004, *p* = 0.002, *p* = 0.017; 72 h: *p* = 0.000, *p* = 0.000, *p* = 0.000). These mutant proteins formed nuclear aggregates that were clearly visible in all the transfected cells. p.R121H was also included since it previously produced nonhomogeneous nuclear staining (48 h: *p* = 0.023; 72 h: *p* = 0.000) [27]. On the contrary, the p.I213V and p.V228M mutant proteins did not have any effect on cell viability (Figure 5A).

At the same time, we evaluated the effect of p.L80F, p.H199Pfs*264 and p.I213Tfs*18 mutant proteins on the mitotic process by immunofluorescence analysis 24 and 72 h after transfection of HeLa cells. Wild type and p.H199Pfs*264 FOXC2 plasmids showed a similar rate of transfection (Figure 5B). Wild type FOXC2-transfected cells maintained the ability to undergo mitosis over time, unlike p.H199Pfs*264 (see Figure 5B). Indeed, 72 h after transfection, the number of wild type FOXC2-transfected cells increased while that of p.H199Pfs*264 decreased. A similar pattern was also detected for p.L80F and p.I213Tfs*18 mutant proteins) Frequent mitotic figures could only be observed in wild type FOXC2-transfected cells at 24 and 72 h (Supplementary Figure S2).

### 2.4. Expression Study of FOXC2 Mutant mRNAs in LD Patients

**Figure 6:**
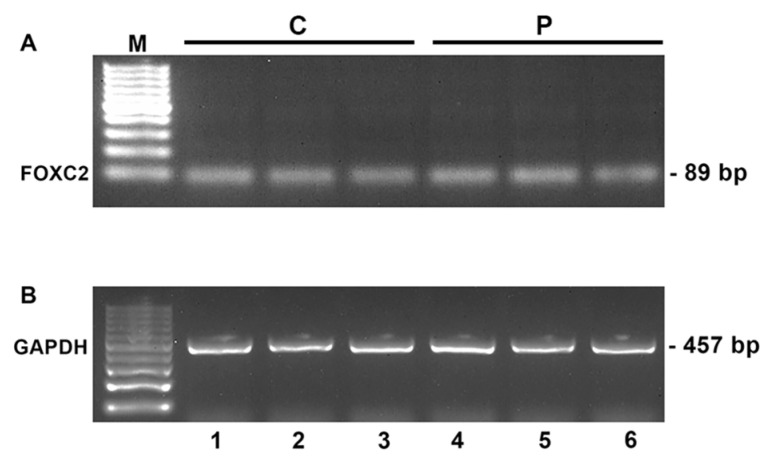
Expression analysis of FOXC2 in peripheral blood cells of LD patients. Gel images show gene expression of FOXC2 (**A**) and GAPDH (**B**), evaluated by comparative RT-PCR. mRNA levels of FOXC2 were similar in control subjects (lanes 1–3) and LD patients (lanes 4–6). M: 100-bp molecular weight marker. C: control subjects; P: patients. Lane 4: LD patient 1 carrying c.595dupC mutation. Lane 5: LD patient 2 carrying c.638delT mutation. Lane 6: LD patient 3 carrying c.638delT mutation.

To verify whether the c.595dupC (p.H199Pfs*264) and c.638delT (p.I213Tfs*18) mutations cause nonsense-mediated decay of mutant mRNAs in vivo, we analyzed total FOXC2 mRNA expression in peripheral blood of LD patients. Comparative RT-PCR analysis detected mRNA in similar amounts in three LD patients carrying frameshift mutations and in control subjects (Figure 6A). RT-PCR analysis of cDNAs obtained without reverse transcriptase (-RT) was also carried out and produced negative results. GAPDH RT-PCR products were used to normalize FOXC2 RT-PCR values for each sample (Figure 6B).

The FOXC2 RT-PCR products obtained from LD patients were then sequenced. FOXC2 mutant mRNAs were detected in the lymphocytes of patients (data not shown). 

### 2.5. Bioinformatic Studies of Mutant Protein Structures 

**Figure 7:**
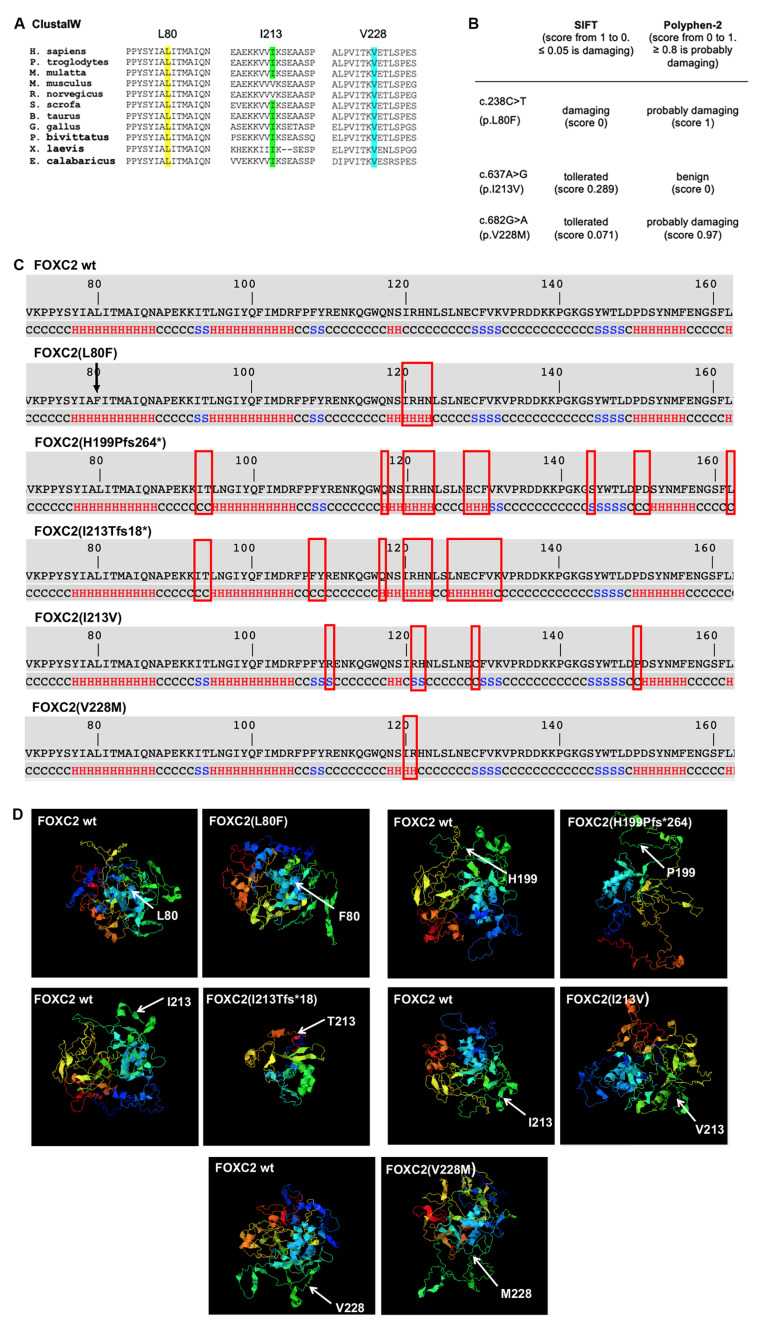
Bioinformatic analysis of FOXC2 mutations. (**A**) The amino acid sequence alignment of FOXC2 in 11 vertebrates (*H. sapiens, P. troglodytes, M. mulatta, M. musculus, R. norvegicus, S. scrofa, B. taurus, G. gallus, P. bivittatus, X. laevis, E. calabaricus*) showed complete conservation of leucine 80 and valine 228 and partial conservation of isoleucine 213; multiple alignments were carried out with Basic GeneBee ClustalW version 2.1, web interface. FOXC2 protein NCBI accession number: NP_005242.1; (**B**) Table reporting SIFT (http://sift.jcvi.org) and PolyPhen-2 (http://genetics.bwh.harvard.edu/pph2) prediction analysis of mutation damage scores for L80F, I213V and V228M mutations; (**C**) forkhead DNA-binding domain (FHD) secondary structure prediction of FOXC2 wild type, p.L80F, p.I213V, p.V228M, p.H199Pfs264* and p.I213Tfs18*. Amino acids 120-123 are predicted to form a coiled-coil structure in the native FOXC2. According to this model, p.L80F (indicated by arrow) causes a change in amino acids 120- 123 from coiled coil to α-helix structure, p.I213V induces a variation from coiled coil to β-strand structure in residues 121-122 and p.V228M modifies amino acids 120 and 121 from coiled coil to α-helix structure. The I213V mutation leads to alteration of other single amino acids: R110 (from coiled coil to β-strand), C130 (from β-strand to coiled coil) and P150 (from α-helix to coiled coil). Frameshift mutations determine many conformational transitions of FHD secondary structure. In particular, in both mutant proteins there are modifications of I93 and T94 (from β-strand to coiled coil), Q117 (from coiled coil to α-helix), I120-N123 (from coiled coil to α-helix), E128 (from coiled coil to α-helix), and C129 and F130 (from β-strand to α-helix). Moreover, H199Pfs264* determines changes in S144 (from coiled coil to β-strand), P150-D151 (from α-helix to coiled coil) and L162 (from α-helix to coiled coil). Finally, variations in F108-Y109 (from β-strand to coiled coil), L126-N127 (from coiled coil to α-helix), V131 (from β-strand to α-helix) and K132 (from β-strand to coiled coil) were observed in p.I213Tfs18*. All changes are indicated with a rectangle; (**D**) 3D models of FOXC2 wild type and mutant proteins. All missense and frameshift mutations show different modifications of protein folding.

The mutations analyzed in this study were localized in the forkhead domain and in the central region of the FOXC2 protein (Figure 1A). To further clarify the pathogenic charge of these mutations, bioinformatic investigations were performed. Multiple alignments, produced by ClustalW, comparing FOXC2 protein sequences identified in eleven vertebrates, showed that p.L80F and p.V228M mutations were located in highly conserved regions (Figure 7A) and may, therefore, affect FOXC2 function.

The analysis carried out using the prediction tools PolyPhen-2 (http://genetics.bwh.harvard.edu/pph2) and SIFT (http://sift.jcvi.org) supported the hypothesis that p.L80F alters FOXC2 activity. Indeed, both programs predicted a deleterious effect of this mutation. p.V228M was only predicted to be damaging by Polyphen-2, while p.I213V was classified as benign or tolerated by both bioinformatic tools (Figure 7B).

To determine whether the missense mutations could cause modifications in FOXC2 conformation, a structural investigation was performed using I-Tasser software. The analysis showed alterations in secondary structure of mutant proteins in the FHD (Figure 7C). The L80F substitution, localized in NLS1, did not seem to modify residues in this region, but it was predicted to cause significant changes in the secondary structure of amino acids between I120 and N123 (from coiled coil to α-helix). Three-dimensional structure prediction analysis showed that these modifications could cause incorrect folding of the mutant protein, decreasing its ability to link to DNA and, consequently, its transcriptional activity (Figure 7D). Although I213 and V228 were located outside the FHD, their substitutions also seemed to produce small modifications in this domain. The 3D models also revealed that both mutant proteins might have an altered tertiary structure (Figure 7D).

In p.H199Pfs264* and p.I213Tfs18*, the frameshift mutations caused a dramatic change in the majority of amino acid sequences. Bioinformatic analysis demonstrated that these modifications could also alter the secondary structure of many amino acids in the FDH domain, increasing the number of residues involved in an α-helix conformation (Figure 7C). These changes could explain the ability of both mutant proteins to strongly bind DNA. Investigation of tertiary structure predicted misfolded states for p.H199Pfs264* and p.I213Tfs18* (Figure 7D). 

## 3. Discussion

FOXC2 transcription factor plays an important role in human pathophysiology, both in genetics and in cancer [19,21,28,29,30,31,32]. So far, about 110 different mutations along the entire gene have been described in LD patients, but little is known about their molecular consequences [4,17,26,27,33]. Collectively, the present data offers new insights into the functional consequences of FOXC2 disease mutations and demonstrates that some mutant proteins may induce cell death.

All mutations but one reduced transcriptional activity (by 30–100%). The mutation p.Y109* is located in the forkhead domain and was predicted to produce a short, truncated protein. Immunofluorescence and immunoblot results clearly showed that p.Y109* was not able to localize into the nucleus, although it still contained the first nuclear localization signal. Therefore, even if it is stably synthesized in vivo, it cannot carry out its transcription factor function. The mutation p.L80F is the first mutation identified in NLS1 of FOXC2. Bioinformatic prediction tools suggested that p.L80F is damaging (Sift) and probably damaging (Polyphen-2). On the basis of homology data comparison, the mutation is in a highly conserved region of the FOXC2 protein. Three-dimensional structure prediction showed that it modifies the folding of the FHD, decreasing its ability to link DNA. In agreement with the bioinformatic data, our functional studies revealed that p.L80F strongly decreased the protein’s capacity to activate the promoter of the reporter gene, although it correctly localized in the nucleus. Indeed, I-Tasser software analysis did not show alterations in NLS1 secondary structure but about twenty amino acids beyond, in the FHD. 

Immunofluorescence analysis of p.L80F mutant proteins shows that they segregate into nuclear aggregates, like other FHD missense mutations already demonstrated to dramatically impair DNA binding activity [26,27,34]. The function of two other missense mutations, p.I213V and p.V228M, both located in the central part of FOXC2 protein, was investigated in this study. The former changes the first amino acid of the consensus synergy (SC) control motif (Ile-Lys-Glu), which is a site for SUMOylation. Danciu et al. demonstrated that the substitution of lysine with arginine at position 214 of FOXC2 (p.K214R) prevented SUMOylation, enhancing protein function. K214R is next to the amino acid change identified in our LD patient [18]. However, our transfection data showed that the p.I213V mutation did not significantly heighten transcriptional ability or modify DNA binding and nuclear localization. SUMOylation might be a new molecular mechanism inducing reversible regulation of FOXC2 expression. However, few data are available on FOXC2 SUMOylation pathways. Moreover, to our knowledge, p.I213V is the first mutation in the SC control motif identified in a patient with LD. The pathogenic role of mutation p.I213V should therefore be carefully evaluated in future clinical studies, also in the light of bioinformatic prediction data that indicated it as benign or tolerated. The last missense mutation is p.V228M, which was predicted as tolerated by SIFT and probably damaging by Polyphen-2. Bioinformatics software resources for molecular diagnosis sometimes provide conflicting indications [27,35] and cannot predict whether a mutation causes a gain or loss of function. The results of our transfection experiments suggested that p.V228M is a pathogenic mutation because it shows a 30% reduction in transcriptional activity with respect to wild type FOXC2. As expected, this mutation does not modify nuclear localization. The impaired transcriptional activity was, therefore, probably due to lower DNA-binding-activity. Accordingly, the 3D model of p.V228M revealed that the mutant protein might have altered tertiary structure due to significant changes in the secondary structure of amino acids located in the FHD (I-Tasser).

Finally, we performed a functional evaluation of two frameshift mutations, p.H199Pfs*264 and p.I213Tfs*18, localized in the central region of FOXC2. They lead to the formation of truncated proteins, lacking more than 50% of the native sequence and with an extra-long stretch of 264 and 18 amino acids, respectively. Although these mutant proteins showed a relevant modification of 3D structure, they conserved the ability to localize to the nucleus and to bind DNA. However, both caused a significant loss of protein function and the formation of nonhomogeneous nuclear aggregates. Indeed, the p.L80F and p.R121H mutations also show nuclear protein aggregates, but they never colocalize with DNA [17,26,27]. Similar results have also been reported for other frameshift and missense FOXC2 mutations [27,34].

When we investigated the consequence of p.H199Pfs*264 and p.I213Tfs*18 protein expression in transfected cells, we found that they strongly induced cell death. Similar results were obtained for missense mutations in the FHD, p.L80F and p.R121H. Conversely, the other missense mutations outside the FHD (p.I213V and p.V228M) did not show protein nuclear aggregation, nor did they alter cell viability. Cells transfected with mutant proteins that cause nuclear aggregation are unable to promote mitotic entry. At different periods after transfection (24, 48 and 72 h), their number did not increase and most of them died. On the contrary, frequent mitotic events can be observed in cells transfected with FOXC2 native sequence, demonstrating that the transcription factor is involved in cell proliferation. This result agrees with a great deal of previously reported evidence attesting the key role of FOXC2 in promoting the proliferation of tumor cells in vitro and in vivo [36,37,38]. It has also been shown that wild type FOXC2 can induce expression of several cell cycle regulators, including cyclin-dependent kinase 1 (CDK1) [19]. CDK1 is a key regulator of each phase of mitosis; indeed, initiation of the mitotic process requires nuclear translocation and activation of CDK1 [39,40]. As described below, we failed to detect mitosis among cells transfected with those FOXC2 mutant plasmids that caused nuclear protein aggregation. Since we did not even observe the initial phases of mitosis (prophase and prometaphase), we hypothesize that these mutations lead to mitotic block from its onset. These findings strengthen the link between FOXC2 and cell proliferation. As the percentage of FOXC2 insertions and deletions (leading to frameshift) is high, and the number of FHD missense mutations identified in LD patients is growing, further studies to assess the molecular consequences of such mutations on cell proliferation would be worthwhile. We do not have any evidence of differences in the stability of mutant FOXC2 mRNAs isolated from lymphocytes of control subjects and LD patients. Our results show that aberrant FOXC2 mRNAs are not eliminated by the nonsense-mediated decay pathway. It therefore cannot be excluded that FOXC2 mutant proteins are synthesized in vivo and exert their harmful effect on the tissues where they are produced. We do not know the molecular mechanism by which FOXC2 evades NMD. FOXC2 apparently falls in the category of an intronless gene that escapes both NMD and other mechanisms that serve to eliminate aberrant transcripts, presumably by virtue of being intronless. This was also demonstrated previously for some other genes [41,42,43].

In conclusion, our functional studies further highlighted the different molecular consequences of some FOXC2 mutations identified in families with lymphedema-distichiasis. We also provided the first evidence that some inactivating mutations cause strong inhibition of cell proliferation. Larger functional and clinical studies are needed to elucidate a possible correlation between different FOXC2 mutations and the broad phenotypic heterogeneity observed in LD patients: heterogeneity that is not yet supported by biochemical or genetic evidence.

## 4. Materials and Methods 

### 4.1. Generation of FOXC2 Site-Directed Mutagenesis Plasmids

FOXC2 cDNA was previously cloned into pcDNA3.1/NT-GFP-TOPO to produce NT-GFP-FOXC2, expressing FOXC2 with GFP at the N-terminus [27]. Point mutations were introduced using the primers reported in Supplementary Table S1 with the Phusion Site-Directed Mutagenesis Kit (Thermo Fisher Scientific, Waltham, MA, USA). After mutagenesis, all expression constructs were verified by DNA sequencing. NT-GFP-FOXC2 (wild type) and NT-GFP-FOXC2 mutant plasmids were used as templates to amplify FOXC2 control and mutant cDNA sequences with FOXC2-F and FOXC2-R primers (reported above). The PCR products were subcloned into pcDNA3.3-TOPO (Invitrogen, Carslbad, CA, USA) to produce control and mutant FOXC2 proteins without any tag.

### 4.2. Analysis of FOXC2 Protein Expression in HeLa Transfected Cells

HeLa cells were transiently transfected with NT-GFP-FOXC2 recombinant plasmids using the Lipofectamine 2000 transfection reagent (Thermo Fisher Scientific Waltham, MA, USA). After 48 h of transfection, cytosol and nucleus subcellular protein fractions were prepared from confluent HeLa cells. After extensive washing with Dulbecco’s phosphate-buffered saline (DPBS), subcellular protein fractions were extracted using FractionPrep cell fractionation kit (Biovision, Milpitas, CA, USA). The protein concentration of cell extracts was quantified by Coomassie (Bradford) Protein Assay Kit (Pierce, Thermo Fisher Scientific, Waltham, MA, USA). Proteins (25 μg/well) were separated by electrophoresis on 10% SDS-polyacrylamide gel (Bio-Rad, Hercules, CA, USA), transferred to a nitrocellulose membrane (Bio-rad, Hercules, CA, USA) and then immunoblotted using a mouse monoclonal antibody (dil. 1:2000) raised against Green Fluorescent Protein (GFP, Abnova, Taipei, Taiwan). Signals were detected using the SuperSignal West Pico Complete Mouse Detection Kit (PIERCE, Thermo Fisher Scientific, Waltham, MA, USA) containing ImmunoPure peroxidase conjugated goat antimouse IgG (dil 1:20,000).

### 4.3. Localization of FOXC2 Mutant Proteins in HeLa and Nuclear Morphological Observation

For transient transfections, HeLa cells were cultured on glass coverslips in Dulbecco’s modified Eagle’s medium supplemented with 10% fetal bovine serum and allowed to adhere overnight. The next day, the cells were transiently transfected with recombinant NT-GFP-FOXC2 plasmids using the Lipofectamine 2000 transfection reagent (Thermo Fisher Scientific, Waltham, MA, USA). After 24 h, cells were fixed, stained with DAPI (4′,6-diamidino-2-phenylindole), and examined with a Leica MB5000B microscope equipped with 40× and 100× Fluorart oil immersion objectives (Leica, Wetzlar, Germany).

Cells transfected with expression vectors containing FOXC2 without tag were permeabilized with phosphate-buffered saline containing 0.1% (*v/v*) Triton X-100 (PBS-X), blocked with 5% (*w/v*) bovine serum albumin in PBS-X and incubated with antiFOXC2 polyclonal antibody (Abcam, ab5060, Cambridge, UK) (1:500) for 1 h at room temperature. After extensive washing in PBS-X, the cells were incubated with a FITC-conjugated goat secondary antibody at a dilution of 1:500 (Thermo Fisher Scientific, Waltham, MA, USA). Cells were stained with DAPI, mounted on slides and examined by microscope as previously described.

HeLa cells were also seeded at 30,000 cells/cm^2^ on coverslips in 3 cm Costar plates, grown to semiconfluence and then transfected with wild type and mutant FOXC2 plasmids for 24 and 72 h. Cells were fixed and stained with DAPI and examined as reported above. 

### 4.4. Luciferase Assays

HeLa cells were cotransfected with 20 ng FOXC1 luciferase reporter [44] and 100 ng wild type or mutant plasmids using TurboFect reagent (Thermo Scientific, Waltham, MA, USA), according to the manufacturer’s recommendations in a 96 well format. Each transfection was set up in triplicate. To measure FOXC2 activity, all transfected cell lines were incubated for 40 h prior to lysis of the culture and addition of substrate from the Britelight plus kit (PerkinElmer, Waltham, MA, USA). Luminescence detection was performed using the Glomax luminometer (Promega, Madison, WI, USA). For each sample, the average expression level in fluorescent units (FU) (from *Photinus pyralis*, reporter) was calculated after correction for transfection efficiency. This was obtained as a measure of GFP fluorescence in the cells transfected with NT-GFP-FOXC2 recombinant plasmids [27]. The same experimental conditions were used for transient transfection of wild type or FOXC2 mutant plasmids subcloned into pcDNA3.3, a mammalian expression vector without tag. Dual-luciferase assays were used to obtain sequential quantification of *Photinus pyralis* luciferase (reporter vector) and *Renilla reniformis* luciferase (control vector). Luminescence detection for all transfected plasmids was performed using the Glomax luminometer (Promega, Madison, WI, USA). Reactions were replicated three times, using the Promega Dual Luciferase Assay kit (Promega, Madison, WI, USA).

### 4.5. Trypan Blue Exclusion Test of Viability

HeLa cells were transfected with wild type and FOXC2 mutant plasmids and incubated at 37 °C in a 95% air/5% CO_2_ atmosphere for 24, 48 and 72 h. The cells were then suspended in DPBS containing trypan blue and examined to determine the percentage of cells with clear cytoplasm (viable cells) versus cells with blue cytoplasm (nonviable cells) under a light microscopic. Each sample was performed in triplicate. The experiment was repeated twice.

### 4.6. RT-PCR Analysis of FOXC2 mRNA from Peripheral Blood of LD Patients

Total RNA was isolated from lymphocytes of LD and control subjects with TRIzol (Invitrogen, Carslbad, CA, USA). All subjects gave their informed consent before they participated in the study. The study was conducted in accordance with the Declaration of Helsinki. One microgram of RNA was converted to cDNA by RT-PCR using random hexamers (0.5 µmg), 400 units of MMLV-RT(Moloney murine leukemia virus), 1.6 mM total dNTPs, 20 units of Rnasin and 0.4 mM dithiothreitol in a 50 µL reaction solution containing 10× RT buffer. Moreover, for each biological sample, 1 µg RNA was incubated with all the reagents reported above with the exception of MMLV-RT.

Before reverse transcription, RNA was treated with DNase I in order to eliminate DNA contamination. RT-PCR reactions were optimized for FOXC2 and GAPDH genes in order to avoid saturation. Regression curves assaying different amounts of cDNAs (corresponding to different mRNA concentrations) and different numbers of amplification cycles were performed (data not shown). Fifty nanograms of cDNA were used to perform PCR amplification with primers designed to produce an 89 bp fragment of the FOXC2 transcript. PCR conditions were as follows: denaturation at 95 °C for 90 s, annealing at 50 °C for 30 s and extension at 72 °C for 30 s in the first round, denaturation at 94 °C for 30 s, annealing at 50 °C for 30 s and extension at 72 °C for 30 s for 24 cycles; denaturation at 94 °C for 30 s, annealing at 50 °C for 30 s and terminal extension at 72 °C for 60 s in the last cycle. Analysis of FOXC2 expression was carried out on +RT and –RT cDNA samples in order to exclude the presence of traces of DNA. Negative control samples were also added to each RT-PCR experiment. The PCR products were electrophoresed on a 2% agarose gel containing GelGreen Nucleic Acid Gel Stain (Biotium, Fremont, CA, USA). The concentration of FOXC2 gene was normalized to the constant level of GAPDH in each sample. Images of gels were acquired (Bio-Rad Gel Doc 2000, Bio-Rad, Hercules, CA, USA) and scanned using Quantity One Analysis software (Bio-Rad, Hercules, CA, USA). This software provides the mean of each band (i.e., the mean intensity of the pixels in the band volume) and the concentration of PCR products, calculated from standards included in each gel.

Finally, the RT-PCR products of LD patients were purified (NucleoSpin Extract II, Macherey-Nagel, Dueren, Germany) and sequenced on a 3730 DNA analyzer by the BigDye Terminator V1.1 Cycle sequencing kit (Applied Biosystems, Foster City, CA, USA).

### 4.7. Statistical and Bioinformatic Analysis

Statistical analysis of quantitative RT-PCR and luciferase assay data was performed using SPSS v.19 package (SPSS, Chicago, IL, USA). Values were compared using Student’s *t*-test. A *p*-value ≤ 0.05 was considered statistically significant.

The effect of amino acid substitutions on protein function was predicted using ClustalW (https://www.ebi.ac.uk/Tools/msa/clustalw2/), SIFT (http://sift.jcvi.org) and PolyPhen (http://genetics.bwh.harvard.edu/pph2) software. A multiple sequence alignment of mammalian FOXC2 proteins was used as input for ClustalW. The NCBI reference sequence (FOXC2 protein NCBI accession number: NP_005242.1.) of human FOXC2 protein was used as input for SIFT and PolyPhen, with default query options. Investigation of secondary and tertiary structure was performed using i-Tasser bioinformatic tool (https://zhanglab.ccmb.med.umich.edu/I-TASSER/).

## Figures and Tables

**Table 1:** Summary of molecular and clinical data of patients.

Patient (Age)	Mutation	Protein Domain	Transcriptional Activity of Mutant Protein *	Age of Onset	Lymphedema	Distichiasis ***	Heart Defects/Other Clinical Findings
1 M (44 y)	L80F	NLS/ FHD	30%	19 y	left foot	no	no
2 M (48 y)	Y109*	FHD	nd	13 y	feet and ankles	yes	varicose veins, cellulitis, spinal extradural cysts
3 F (53 y)	H199Pfs*264	Central region	28%	27 y	right foot	yes	varicose veins,
							cardiac arrhythmia,
							upslanting toenails
4 F (39 y)	I213V	Central region	103%	28 y	left foot	no	no
5 F (54 y)	I213Tfs*18	Central region	30%	12 y	both feet	yes	no
6 F (8 y)	V228M	Central region	70%	congenital	right foot	no	no

* The percentage of transcriptional activity of FOXC2 mutant proteins was calculated taking FOXC2 wild type signal as 100% (Luciferase reporter assay). *** Patients were examined for evidence of distichiasis by an ophthalmologist using a slit lamp. M: Male; F: Female.

## Supplementary Materials

Supplementary materials can be found at https://www.mdpi.com/1422-0067/21/14/5112/s1.

## Abbreviations

FOXC2	Forkhead transcription factor C2
LD	Lymphedema-distichiasis
EMT	Epithelial-mesenchymal transition
AD-1	Transactivation domain 1
FHD	Forkhead DNA binding domain
NLS1	Nuclear localization signal 1
NLS2	Nuclear localization signal 2
AD-2	Transactivation domain 2
ID2	Inhibitory domain 2
PTC	Premature termination codon
NMD	Nonsense-mediated decay
SC	Consensus synergy
CDK1	Cyclin-dependent kinase 1
DPBS	Dulbecco’s phosphate-buffered saline
GFP	Green fluorescent protein
FU	Fluorescent Units
MMLV-RT	Moloney murine leukemia virus

## References

[1] Fang J., Dagenais S.L., Erickson R.P., Arlt M.F., Glynn M.W., Gorski J.L., Seaver L.H., Glover T.W. (2000). Mutations in FOXC2 (MFH-1), a forkhead family transcription factor, are responsible for the hereditary Lymphedema-Distichiasis Syndrome. Am. J. Hum. Genet..

[2] Bell R., Brice G., Child A.H., Murday V.A., Mansour S., Sandy C.J., Collin J.R.O., Brady A.F., Callen D.F., Burnand K. (2001). Analysis of lymphedema-distichiasis families for FOXC2 mutations reveals small insertions and deletions throughout the gene. Hum. Genet..

[3] Brice G., Mansour S., Bell R., Collin J.R.O., Child A.H., Brady A.F., Sarfarazi M., Burnand K.G., Jeffery S., Mortimer P. (2002). Analysis of the phenotypic abnormalities in Lymphoedema-distichiasis syndrome in 74 patients with FOXC2 mutations or linkage to 16q24. J. Med. Genet..

[4] Finegold D.N., Kimak M.A., Lawrence E.C., Levinson K.L., Cherniske E.M., Pober B.R., Dunlap J.W., Ferrel R.E. (2001). Truncating mutations in FOXC2 cause multiple lymphedema syndromes. Hum. Mol. Genet..

[5] Erikson R.P., Dagenais S.L., Caulder M.S., Downs C.A., Herman G., Jones M.C., Kerstiens-Frederikse W.S., Lidral A.C., McDonalds M., Nelson C.C. (2001). Clinical heterogeneity in lymphedema-distichiasis with FOXC2 truncating mutations. J. Med. Genet..

[6] Sabine A., Petrova T.V. (2014). Interplay of mechanotransduction, FOXC2, connexins, and calcineurin signaling in lymphatic valve formation. Adv. Anat. Embryol. Cell. Biol..

[7] Mani S.A., Guo W., Liao M.J., Eaton E.N., Ayyanan A., Zhou A.Y., Brooks M., Reinhard F., Zhang C.C., Shipitsin M. (2008). The epithelial-mesenchymal transition generates cells with properties of stem cells. Cells.

[8] Mani S.A., Yang J., Brooks M., Schwaninger G., Zhou A., Miura N., Kutok J.L., Hartwell K., Richardson A.L., Weinberg R.A. (2007). Mesenchyme Forkhead 1 (FOXC2) plays a key role in metastasis and is associated with aggressive basal-like breast cancers. Proc. Natl. Acad. Sci. USA.

[9] Hollier B.G., Tinnirello A.A., Werden S.J., Evans K.W., Taube J.H., Sarkar T.R., Sphyris N., Shariati M., Kumar S.V., Battula V.L. (2013). FOXC2 expression links epithelial-mesenchymal transition and stem cell properties in breast cancer. Cancer Res..

[10] De Craene B., Berx G. (2013). Regulatory networks defining EMT during cancer initiation and progression. Nat. Rev. Cancer..

[11] Cui Y.M., Jiao H.L., Ye Y.P., Chen C.M., Wang J.X., Tang N., Li T.T., Lin J., Qi L., Wu P. (2015). FOXC2 promotes colorectal cancer metastasis by directly targeting MET. Oncogene.

[12] Paranjape A.N., Soundararajan R., Werden S.J., Joseph R., Taube J.H., Liu H., Rodriguez-Canales J., Sphyris N., Wistuba I., Miura N. (2016). Inhibition of FOXC2 restores epithelial phenotype and drug sensitivity in prostate cancer cells with stem-cell properties. Oncogene.

[13] Yang C., Cui X., Dai X., Liao W. (2016). Downregulation of Foxc2 enhances apoptosis induced by 5-fluorouracil through activation of MAPK and AKT pathways in colorectal cancer. Oncol. Lett..

[14] Li C., Ding H., Tian J., Wu L., Wang Y., Xing Y., Chen M. (2016). Forkhead box protein C2 (FOXC2) promotes the resistance of human ovarian cancer cells to cisplatin in vitro and in vivo. Cell. Physiol. Biochem..

[15] Hayashi H., Sano H., Seo S., Kume T. (2008). The Foxc2 transcription factor regulates angiogenesis via induction of integrin beta3 expression. J. Biol. Chem..

[16] Isogai C., Laug W.E., Shimada H., Declerck P.J., Stins M.F., Durden D.L., Erdreich-Epstein A., DeClerck Y.A. (2001). Plasminogen activator inhibitor-1 promotes angiogenesis by stimulating endothelial cell migration toward fibronectin. Cancer Res..

[17] Berry F.B., Tamini Y., Carle M.V., Lehmann O.J., Walter M.A. (2005). The establishment of a predictive mutational model of the forkhead domain through the analyses of FOXC2 missense mutations identified in patients with hereditary lymphedema with distichiasis. Hum. Mol. Genet..

[18] Danciu T.E., Chupreta S., Cruz O., Fox J.E., Whitman M., Iñiguez-Lluhí J.A. (2012). Small ubiquitin-like modifier (SUMO) modification mediates function of the inhibitory domains of developmental regulators FOXC1 and FOXC2. J. Biol. Chem..

[19] Ivanov K.I., Agalarov Y., Valmu L., Samuilova O., Liebl J., Houhou N., Maby-El Hajjami H., Norrmén C., Jaquet M., Miura N. (2013). Phosphorylation regulates FOXC2-mediated transcription in lymphatic endothelial cells. Mol. Cell. Biol..

[20] Lam E.W.F., Brosens J.J., Gomes A.R., Koo C.Y. (2013). Forkhead box proteins: Tuning forks for transcriptional harmony. Nature.

[21] Bahuau M., Houndayer C., Tredano M., Soupre V., Couderc R., Vazquez M.P. (2002). FOXC2 truncating mutation in distichiasis, lymphedema, and cleft palate. Clin. Genet..

[22] Brouillard P., Boon L., Vikkula M. (2014). Genetics of lymphatic anomalies. Clin. Investig..

[23] Michelini S., Vettori A., Maltese P.E., Cardone M., Bruson A., Fiorentino A., Cappellino F., Sainato V., Guerri G., Marceddu G. (2016). Genetic Screening in a Large Cohort of Italian Patients Affected by Primary Lymphedema Using a Next Generation Sequencing (NGS) Approach. Lymphology.

[24] Beißel C., Grosse S., Krebber H. (2020). Dbp5/DDX19 Between Translational Readthrough and Nonsense Mediated Decay. Int. J. Mol. Sci..

[25] Michelini S., Degiorgio D., Cestari M., Corda D., Ricci M., Cardone M., Mander A., Famoso L., Contini E., Serrani R. (2012). Clinical and Genetic Study of 46 Italian Patients with Primary Lymphedema. Lymphology.

[26] Van Steensel M.A., Damstra R.J., Heitink M.V., Bladergroen R.S., Veraart J., Steijlen P.M., van Geel M. (2009). Novel missense mutations in the FOXC2 gene alter transcriptional activity. Hum. Mutat..

[27] Tavian D., Missaglia S., Maltese P.E., Michelini S., Fiorentino A., Ricci M., Serrani R., Walter M.A., Bertelli M. (2016). FOXC2 disease-mutations identified in lymphedema-distichiasis patients cause both loss and gain of protein function. Oncotarget.

[28] Norrmén C., Ivanov K.I., Cheng J., Zangger N., Delorenzi M., Jaquet M., Miura N., Puolakkainen P., Horsley V., Hu J. (2009). FOXC2 controls formation and maturation of lymphatic collecting vessels through cooperation with NFATc1. J. Cell Biol..

[29] Wang T., Zheng L., Wang Q., Hu Y.W. (2018). Emerging Roles and Mechanisms of FOXC2 in Cancer. Clin. Chim. Acta.

[30] Kume T., Shackour T. (2018). Meta-analysis of the likelihood of FOXC2 expression in early- and late-stage tumors. Oncotarget.

[31] Welsh J.D., Hoofnagle M.H., Bamezai S., Oxendine M., Lim L., Hall J.D., Yang J., Schultz S., Engel J.D., Kume T. (2019). Hemodynamic regulation of perivalvular endothelial gene expression prevents deep venous thrombosis. J. Clin. Investig..

[32] Wang Y.Q., Xu Z.M., Wang X.L., Zheng J.K., Du Q., Yang J.X., Zhang H.C. (2020). LncRNA FOXC2-AS1 regulated proliferation and apoptosis of vascular smooth muscle cell through targeting miR-1253/FOXF1 axis in atherosclerosis. Eur. Rev. Med. Pharmacol. Sci..

[33] Sholto-Douglas-Vernon C., Bell R., Brice G., Mansour S., Sarfarazi M., Child A.H., Smith A., Mellor R., Burnand K., Mortimer P. (2005). Lymphoedema-distichiasis and FOXC2: Unreported mutations, de novo mutation estimate, families without coding mutations. Hum. Genet..

[34] Zhang L., He J., Han B., Lu L., Fan J., Zhang H., Ge S., Zhou Y., Jia R., Fan X. (2016). Novel *FOXC2* Mutation in Hereditary Distichiasis Impairs DNA-Binding Activity and Transcriptional Activation. Int. J. Biol. Sci..

[35] Pennisi E.M., Missaglia S., Di Mauro S., Akman H.O., Tavian D. (2015). A myopathy with unusual features caused by PNPLA2 gene mutations. Muscle Nerve.

[36] Yang F., Lv L., Zhang K., Cai Q., Liu J., Jiang Y. (2017). Elevated FOXC2 Expression Promotes Invasion of HCC Cell Lines and is Associated with Poor Prognosis in Hepatocellular Carcinoma. Cell. Physiol. Biochem..

[37] Zhang W., Zhang X., Li J., Zheng J., Hu X., Xu M., Mao X., Ling J. (2018). Foxc2 and BMP2 Induce Osteogenic/Odontogenic Differentiation and Mineralization of Human Stem Cells from Apical Papilla. Stem Cells Int..

[38] Zhang C., Li H., Guo X. (2019). FOXC2-AS1 regulates phenotypic transition, proliferation and migration of human great saphenous vein smooth muscle cells. Biol. Res..

[39] Petronczki M., Lénárt P., Peters J.M. (2008). Polo on the Rise-from Mitotic Entry to Cytokinesis With Plk1. Dev. Cell.

[40] Terhune S.S., Jung Y., Cataldo K.M., Dash R.K. (2020). Network Mechanisms and Dysfunction Within an Integrated Computational Model of Progression Through Mitosis in the Human Cell Cycle. PLoS Comput. Biol..

[41] Miura N., Iida K., Kakinuma H., Yang X.L., Sugiyama T. (1997). Isolation of the Mouse (MFH-1) and Human (FKHL 14) Mesenchyme Fork head-1 Genes Reveals Conservation of Their Gene and Protein Structures. Genomics.

[42] Brocke K.S., Neu-Yilik G., Gehring N.H., Hentze M.W., Kulozik A.E. (2002). The Human Intronless Melanocortin 4-receptor Gene Is NMD Insensitive. Hum. Mol. Gen..

[43] Dyle M.C., Kolakada D., Cortazar M.A., Jagannathan S. (2020). How to Get Away With Nonsense: Mechanisms and Consequences of Escape From Nonsense-Mediated RNA Decay. Wiley Interdiscip. Rev. RNA.

[44] Saleem R.A., Banerjee-Basu S., Berry F.B., Baxevanis A.D., Walter M.A. (2001). Analyses of the effects that disease-causing missense mutations have on the structure and function of the winged-helix protein FOXC1. Am. J. Hum. Genet..

