# Supplementary material for: FOXC2 Disease Mutations Identified in Lymphedema Distichiasis Patients Impair Transcriptional Activity and Cell Proliferation
Source: Int J Mol Sci. 2020 Jul 20;21(14):5112. doi: 10.3390/ijms21145112 (PMC7404146; doi:10.3390/ijms21145112)
Supplement: Supplementary file 1 [file ijms-21-05112-s001.pdf]

Supplementary Table 1

Primer name	Sequence
FOXC2(L80F)-F	ACAGCTACATCGCGTTCATCACCATGGCC
FOXC2(L80F)-R	GGCCATGGTGATGAACGCGATGTAGCTGT
FOXC2(Y109*)-F	CGCTTCCCCTTCTAACGGGAGAACAAGCAG
FOXC2(Y109*)-R	CTGCTTGTTCTCCCGTTAGAAGGGGAAGCG
FOXC2(H199Pfs264*)-F	CGGCCACCCCCCCACCTAGCGGAC
FOXC2(H199Pfs264*)-R	GTCCGCTAGGTGGGGGGGTGGCCG
FOXC2(I213V)-F	AGAAGAAGGTGGTGGTCAAGAGCGAGGCGG
FOXC2(I213V)-R	CCGCCTCGCTCTTGACCACCACCTTCTTCT
FOXC2(I213Tfa18*)-F	GAAGAAGGTGGTGACAAGAGCGAGGCGG
FOXC2(I213Tfa18*)-R	CCGCCTCGCTCTTGTCACCACCTTCTTC
FOXC2(V228M)-F	CGGTCATCACCAAGATGGAGACGCTGAGC
FOXC2(V228M)-R	GCTCAGCGTCTCCATCTTGGTGATGACCG

Supplementary Figure 1

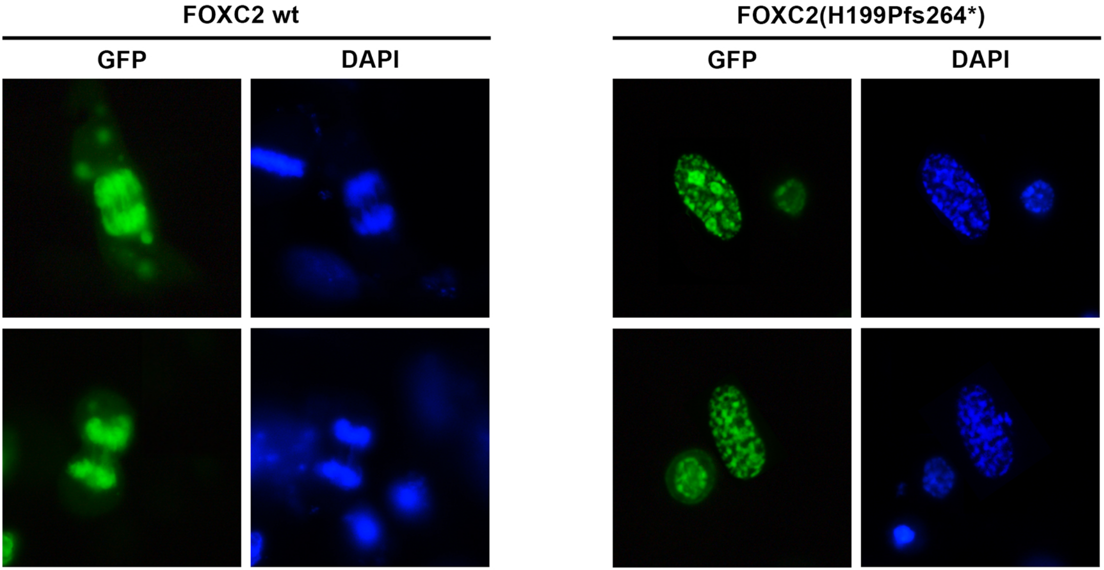

Supplementary Figure 1. Mitotic detection in HeLa cells transiently transfected with *FOXC2*-GFP and *FOXC2*(H199Pfs264*)-GFP plasmid
Immunofluorescence analysis shows a high number of mitotic cells, 72 h after transient transfection with *FOXC2*-GFP plasmid (left panel). On the contrary, severe decrease of mitosis and abnormal nuclear morphology is detected at 72 h post-transfection with *FOXC2*(H199Pfs264*)-GFP plasmid (right panel). Fluorescence of *FOXC2*-GFP recombinant proteins were in green. DAPI was used to stain nuclei (in blue). Images were at 40X magnification.
